# Profiling of immune cell subsets and functional characteristics of cervical cancer based on single cell RNA sequencing

**DOI:** 10.3389/fimmu.2025.1658705

**Published:** 2025-09-25

**Authors:** Yue Yuan, Dejun Sun, Mingyue Yang, Ying Xia, Chunli Yuan, Xiaosong Wang

**Affiliations:** ^1^ Department of Regenerative Medical Science, School of Pharmaceutical Sciences, Jilin University, Changchun, China; ^2^ Second Department of General Gynecology, Gynecologic and Obstetrics Center, The First Hospital of Jilin University, Changchun, China; ^3^ Institute of Translational Medicine, The First Hospital of Jilin University, Changchun, China

**Keywords:** cervical cancer, single-cell RNA sequencing, immune cell, tumor microenvironment, CCR7

## Abstract

**Introduction:**

A comprehensive characterization of immune cells within the tumor microenvironment (TME) of cervical squamous cell carcinoma (CSCC) is essential to advance understanding of tumor biology and guide immunotherapy development.

**Methods:**

Using single-cell RNA sequencing, we analyzed 14,441 immune cells isolated from tumor tissues and paratumor tissues of three CSCC patients. By integrating data on tumor suppressors, oncogenic factors, cytokines, and chemokines, we performed differential gene expression analyses across immune populations.

**Results:**

This analysis identified nine major immune cell subsets, including CD8^+^ T cells, regulatory T cells (Tregs), natural killer/T cells (NK/T cells), B cells, plasmacytoid dendritic cells (pDCs), and macrophages. Notably, elevated CCR7 expression in exhausted CD8^+^ T cells was associated with improved prognosis, suggesting it as a preliminary candidate for an immunoregulatory target that warrants further investigation. Additionally, CCL5 levels were elevated in Tregs, indicating a possible involvement in their recruitment to the tumor site that requires further validation. Furthermore, differential gene expression analysis identified candidate genes for further mechanistic investigation.

**Discussion:**

This systematic comparison of the TME between tumor and paratumor tissues reveals dynamic changes in the immune landscape, providing insights and suggesting potential targets for further study to enhance understanding and treatment of CSCC.

## 1 Introduction

Cervical cancer (CC) ranks as the fourth most common malignancy among women worldwide, following breast, colorectal, and lung cancers (1). In some low- and middle-income countries, it is the second most prevalent cancer in women (2). Global data report approximately 604,000 new cases and over 34,200 deaths annually (3, 4). This highlights the urgent need to clarify CC pathogenesis and advance novel therapeutic approaches.

CC progression is driven by both cancer cell-intrinsic factors and the dynamic, heterogeneous tumor microenvironment (TME) (5). Immune cell spatial distribution and function within the TME critically influence tumor outcomes. For instance, higher cytotoxic T cell infiltration correlates with improved patient survival (6, 7), whereas regulatory T cells (Tregs) facilitate immune evasion via immunosuppression (5). B cells play dual roles, promoting tumor growth through pro-angiogenic factor secretion (8, 9) and supporting antitumor immunity via antigen presentation and antibody production (10, 11). Natural killer (NK) cells directly kill tumor cells (12–14). The mononuclear phagocyte system—including monocytes, macrophages, and dendritic cells—mediates innate immune recognition and pathogen clearance while linking to adaptive immunity by presenting antigens to T cells (15).

Advances in single-cell RNA sequencing (scRNA-seq) have transformed the analysis of TME complexity, yet studies on CC TME remain limited (16, 17). Prior work largely examines TME dynamics during CC progression or gene expression linked to chemoresistance (18, 19), with few direct comparisons of immune infiltration between tumor and adjacent non-tumor tissues. Here, we applied scRNA-seq to paired tumor and paratumor tissues from newly diagnosed CC patients, minimizing inter-individual variability and enhancing data robustness. We characterized gene expression profiles of key immune populations, including CD8^+^ T cells, Tregs, B cells, NK/T cells, plasmacytoid dendritic cells (pDCs), and macrophages. This study aims to advance understanding of the immune landscape in CC TME and provide a foundation for future research. Moreover, the generated scRNA-seq dataset represents a valuable resource for investigating immune cell biology.

## 2 Materials and methods

### 2.1 Patients and samples

Fresh squamous cervical cancer specimens were collected from three patients undergoing primary surgery at the Department of Obstetrics and Gynecology, First Hospital of Jilin University, between June 2019 and August 2020. None received preoperative treatment. Patient and sample details are listed in **Table 1**. Ages ranged from 44 to 64 years, with grade 1, FIGO stage IB1 tumors, and no lymph node metastasis. Tumor and paratumor tissues were promptly harvested post-excision. Single-cell RNA sequencing (scRNA-seq) was performed on 14,812 tumor cells and 16,190 paratumor cells.

**Table 1:** The clinical characteristics of the samples.

Clinico-pathological parameter	Patient 1	Patient2	Patient3
Age	51	44	64
FIGO stage	IB1	IB1	IB1
TNM stage	T2a1N0	T1b1N0	T1b1N0
Lymth nodes	Negative	Negative	Negative
Tummor size (mm)^3^	35*28*25	32*25*17	40*35*12
Vaso-invasion	Present	Present	Absent
Infiltration depth	1/2	>2/3	>2/3
HPV type	/	/	16
Cell number (Tumor/Paratumor)	5684/7276	4677/3657	4451/5257

Tumor: cervical cancer tissues; Paratumor: para-tumor tissues.

### 2.2 Tissue processing

Samples of cervical squamous cell carcinoma (CSCC), both tumor and paratumor, were procured from the tumor mass and regions located 1 cm away from the tumor boundary, respectively. The tissue samples were washed with PBS (CORNING) before being stored in MACS Tissue Storage Solution (Miltenyi Biotec). Subsequently, they were processed into single-cell suspensions.

### 2.3 Single-cell RNA sequencing

**Figure 1:**
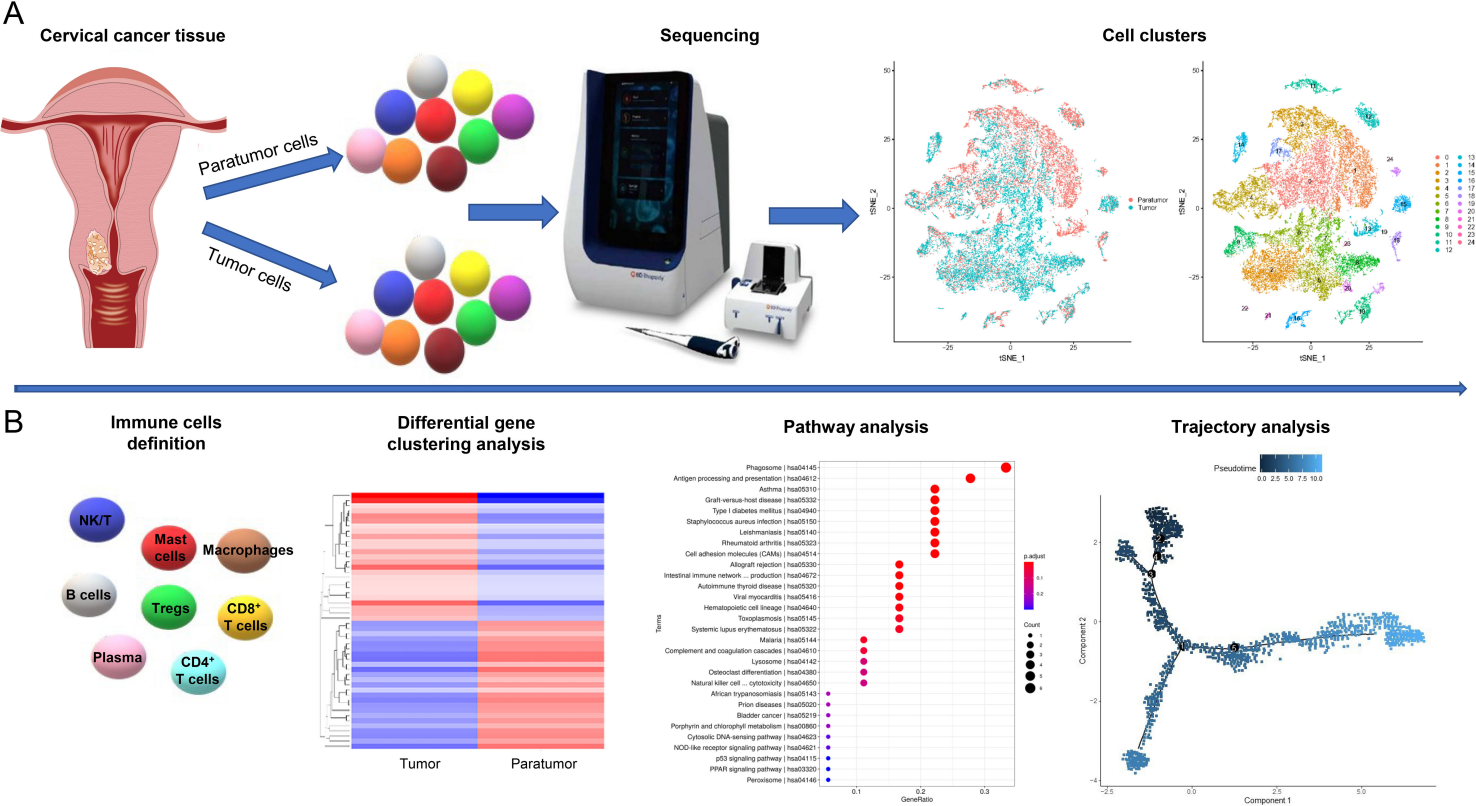
Study Design Overview. **(A)** Single-cell transcriptomic profiling of tumor and paratumor tissues was performed using the BD Resolve single-cell whole transcriptome amplification workflow. Unsupervised clustering identified 25 distinct cell populations. **(B)** Immune cell clusters were characterized using an integrated computational framework combining differential gene expression, pathway analysis, and developmental trajectory reconstruction.

The cell suspension was processed on the Rhapsody™ Cartridge (BD) as per manufacturer’s protocol to produce single-cell magnetic beads within microwells. Subsequently, the captured cells were lysed, and their RNA was barcoded via reverse transcription within individual microwells. The resultant cDNA was amplified and its quality evaluated using the Agilent 4200 instrument. Whole transcriptome libraries were created following the BD Resolve single cell whole transcriptome amplification workflow. These libraries underwent sequencing using an Illumina Novaseq6000 sequencer, achieving a minimum depth of 50,000 reads per cell employing a pair-end 150 bp (PE150) reading strategy. The relevant cell populations were further analyzed using 3’mRNA single-cell transcriptome sequencing (scRNA-seq) incorporating scRNA-seq barcoding and sequencing methodologies (**Figure 1A**). Detailed metrics from the barcoding and sequencing processes of scRNA-seq are presented in **Supplementary Table S1**.

### 2.4 Data analysis

The Resolve analysis pipeline was employed to process sequencing data (fastq files). Initially, raw data in the form of fastq reads were processed to derive clean data, referred to as clean reads. Subsequent analyses relied on this high-quality clean data. The p-values were adjusted using the Benjamini and Hochberg’s approach to control the false discovery rate (FDR). Genes with |avg_logFC|>0 and p-value < 0.05 were identified as differentially expressed. The Seurat 3.0 R package was utilized for the identification of differentially expressed genes (DEGs) and cell clustering. A comprehensive functional analysis, including GO, KEGG, Reactome, and Disease enrichment, was conducted on the top 20 DEGs within each cluster. Protein-protein interactions were sourced from the STRING database with a combined score of ≥700. Predictions for transcription factors were made within 2000 bp upstream and 500 bp downstream of the transcription start site (TSS) for the top 25 marker genes of each cluster, using TFBS Tools and the JASPAR database. GSEA was executed using the GSEA software version 2.2.2.4, incorporating predefined gene sets from the Molecular Signatures Database (MSig DB v6.2). Single-cell trajectories were constructed using the Monocle R package.

The trajectory plot was generated through a multi-step process. Initially, cells exhibiting extremely low or high total mRNA counts were excluded based on the overall distribution, retaining only those within an intermediate range to eliminate non-single-cell contaminants. The filtered gene expression data were then assessed to confirm an approximate log-normal distribution, ensuring analytical robustness. Genes were selected based on the following criteria: expression in at least 10 cells, average expression value > 0.1, q-value < 0.01 from differential expression analysis, and dispersion value greater than or equal to the expected dispersion value. Subsequently, the multidimensional expression profiles of these genes were reduced to two dimensions, and cells were ordered to reconstruct the cellular trajectory.

We performed a retrospective in silico analysis of the Cervical Squamous Cell Carcinoma and Endocervical Adenocarcinoma (CESC) cohort from The Cancer Genome Atlas (TCGA). Raw RNA-sequencing counts (STAR-aligned) were obtained via the TCGAbiolinks R package (v2.28.0) and converted into a SummarizedExperiment object. After removing non-coding and duplicate genes—retaining the transcript with the highest variance—tumor-only samples (barcode suffix “-01A”) were selected. Gene-level TPM (transcripts per million) values were calculated using gene lengths from GENCODE v22 following the formula TPM = (reads_per_gene/gene_length_kb)/(total_reads_per_sample/1e6). Immune cell fractions for 22 subtypes were inferred from the tumor TPM matrix using the CIBERSORT algorithm with the LM22 signature.

Clinical follow-up data, including overall survival (OS) time and vital status, were sourced from the TCGA-CESC clinical supplement and harmonized by 12-character patient IDs. Samples missing OS data were excluded. Cox proportional hazards regression was applied to assess associations between inferred immune cell subtypes and OS, adjusting for age at diagnosis, FIGO stage, tumor grade, and relevant covariates. Variables with a single level were omitted. Hazard ratios (HRs) with 95% confidence intervals and two-sided Wald p-values were reported, with significance defined as p < 0.05. All analyses were conducted in R version 4.4.3 (R Foundation, Vienna, Austria).

### 2.5 Online prediction software

We used Cellmarker (http://biocc.hrbmu.edu.cn/CellMarker) (20) and Panglao DB (https://panglaodb.se/) (21) to identify the majority of immune cell types. Metascape (https://metascape.org/gp/index.html) was used for gene enrichment analysis and protein-protein interaction analysis (22). We used GEPIA2 (http://gepia2.cancer-pku.cn/#index) (23, 24)to investigate the correlations between gene expression levels and the prognosis of cervical squamous cell cancer. The Open Targets software enables us to analyze the pathways and diseases associated with CCR7 (https://www.opentargets.org/) (25). Tumor Immune Estimation Resource (TIMER) database (https://cistrome.shinyapps.io/timer/) was used to identify the correlation between CCR7 and immune cells in cervical cancer (26). We utilized STRING (https://string-db.org/cgi/input.pl) to create an interaction network between CCL5 and other important proteins (27). CancerSEA (http://biocc.hrbmu.edu.cn/CancerSEA/) was used to explore the potential roles of the genes in cancer (28). The Cistrome DB Toolkit database (http://dbtoolkit.cistrome.org) was used to analyze the top 20 differentially expressed TFs that might regulate CCL5 in cancers (29). GEPIA2 (http://gepia2.cancer-pku.cn/#index) facilitates the analysis of differential gene expression, correlation analysis, and dimensionality reduction analysis (23).

### 2.6 Statistical analysis

Data were presented using the mean ± standard error of the mean (SEM) format. The GraphPad Prism software, version 10.0 (GraphPad Software, San Diego, CA, USA), was employed for both statistical analyses and graphical presentations. A t-test was administered when a normality test was successfully passed; otherwise, the nonparametric Mann-Whitney test was utilized. Correspondingly, either the Pearson or the nonparametric Spearman method was applied for correlation analyses. In terms of cut-off values, P < 0.05 was deemed statistically significant.

### 2.7 Ethics approval and consent to participate

The study was approved by the Institutional Medical Ethics Review Board of the First Hospital of Jilin University in compliance with the Declaration of Helsinki, the reference number was 2019-320.

## 3 Results

### 3.1 Acquisition of scRNA-seq profiles and cell clustering

We conducted scRNA-seq on six samples, including three pairs of CSCC tissues and their corresponding paratumor tissues from three patients. Detailed sample information is available in **Table 1**. To thoroughly analyze the tumor microenvironments, we employed unsupervised clustering on all cells, identifying cell clusters based on their molecular and functional characteristics (**Figure 1A**). We then classified the immune cell subtypes and identified their key factors. Using the top 25 upregulated and 25 downregulated genes, we conducted heat map and pathway analyses to illustrate the developmental trajectories of the cells (**Figure 1B**). Finally, we validated the expression of specific genes and examined their correlations with clinical features.

### 3.2 Single cell RNA-seq reveals the immune landscape of human CSCC

**Figure 2:**
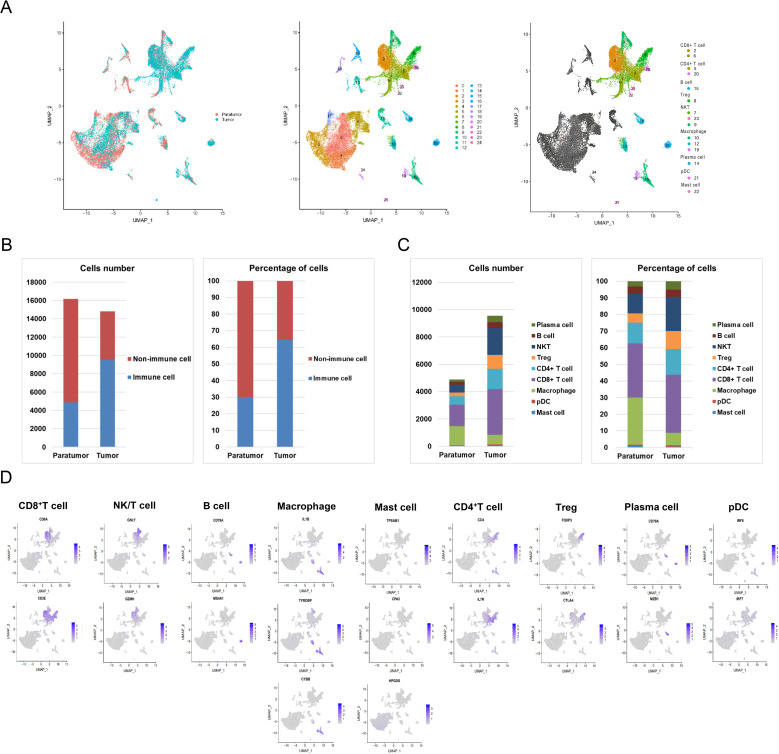
Spatial Distribution of Immune Cell Subsets. **(A)** Uniform Manifold Approximation and Projection (UMAP) visualization of major immune lineages identified via graph-based clustering. **(B)** Bar chart showing the counts and proportions of immune and non-immune cells in paratumor and tumor tissues. **(C)** Detailed quantification and proportion of all immune cell types in paratumor and tumor tissues. **(D)** Expression of cell-type-specific marker genes mapped onto the UMAP plot.

Following initial quality control, we retained a total of 31,002 cells, which were categorized into 25 distinct clusters visualized in a two-dimensional UMAP map (**Figure 2A**). Clusters were defined using cell marker databases and literature references (16, 18, 20, 21, 30). Among these, fifteen clusters were identified as immune cells. The UMAP plots illustrated the expression of cell-type-specific marker genes. Clusters 2 and 6, characterized by high levels of CD3E and CD8A, were classified as CD8^+^ T cells. Clusters 7, 9, and 23 exhibited elevated levels of GNLY and GZMH, identifying them as NK/T cells. Cluster 15 was recognized as B cells based on CD79A and MS4A1 expression. Macrophages were assigned to clusters 10, 12, and 19 due to high expression of IL1B, TYROBP, and CYBB. Cluster 22 was identified as mast cells owing to the selective expression of TPSAB1, CPA3, and HPGDS. Clusters 5 and 20 were designated as CD4^+^ T cells based on CD4 and IL7R expression. Cluster 8 showed significant expression of FOXP3 and CTLA4, classifying it as Tregs. Cluster 14 was annotated as plasma cells due to its expression of CD79A and MZB1. Finally, cluster 21 was classified as pDC cells based on elevated IRF8 and IRF7 levels (**Figures 2A, D**).

As shown in **Figure 2B** and **Supplementary Table S2**, the total number of immune cells was significantly greater in tumor tissue (9,560 cells) than in paratumor tissue (4,881 cells). Quantitative analysis indicated that immune cells comprised 30.15% (4,881/16,190) of paratumor tissue, while they represented 64.54% (9,560/14,812) of the cellular makeup in tumor tissue. Further comparative analysis, presented in **Figure 2C** and **Supplementary Table S3**, revealed distinct differences in the distribution of specific immune cell subsets between tumor and paratumor tissues. Paratumor tissue had a higher number of macrophages (1,382 vs. 701) and mast cells (44 vs. 39) compared to tumor tissue. In contrast, tumor tissue exhibited increased populations of plasma cells (480 vs. 157), B cells (421 vs. 215), NK/T cells (1,964 vs. 574), Tregs (1,025 vs. 270), CD4^+^ T cells (1,489 vs. 612), CD8^+^ T cells (3,346 vs. 1,586), and pDC cells (95 vs. 41). Notably, while the quantity of B cells showed slight variation between tumor and paratumor tissues, their percentage remained equal.

To elucidate the prognostic impact of tumor-infiltrating immune cells in cervical cancer (CC) patients, we performed Cox proportional hazards regression analysis using the TCGA-CESC dataset, which includes cervical squamous cell carcinoma and endocervical adenocarcinoma (CESC). The analysis assessed the association between immune cell infiltration and postoperative outcomes, including recurrence, cancer-related death, or survival time. Memory B cells (HR = 6.51E-51, p = 0.0375), follicular helper T cells (HR = 1.04E-33, p = 0.034868), CD8^+^ T cells (HR = 1.94E-122, p = 4.1E-5), M2 macrophages (HR = 2.66E-78, p = 0.000473), activated NK cells (HR = 2.26E-80, p = 0.043992), activated dendritic cells (HR = 1.14E-31, p = 0.034763), and activated mast cells (HR = 6.78E-101, p = 8.9E-4) were significantly associated with favorable prognosis. In contrast, regulatory T cells (Tregs) (HR = 1.71E+35, p = 0.0280), γδ T cells (HR = 1.76E+33, p = 0.012083), resting NK cells (HR = 1.05E+115, p = 0.004688), eosinophils (HR = 2.92E+47, p = 0.004435) and M0 macrophages (HR = 2.09E+47, p = 0.002562) correlated with poor outcomes. naive B cells, Plasma cells, naive CD4^+^ T cells, resting and activated memory CD4^+^ T cells, monocytes, M1 macrophages, resting dendritic cells, resting mast cells, and neutrophils showed no significant prognostic relevance (p > 0.05).

### 3.3 Elevated CCR7 expression in tumor-resident exhausted CD8^+^ T cells relative to paratumoral counterparts

**Figure 3:**
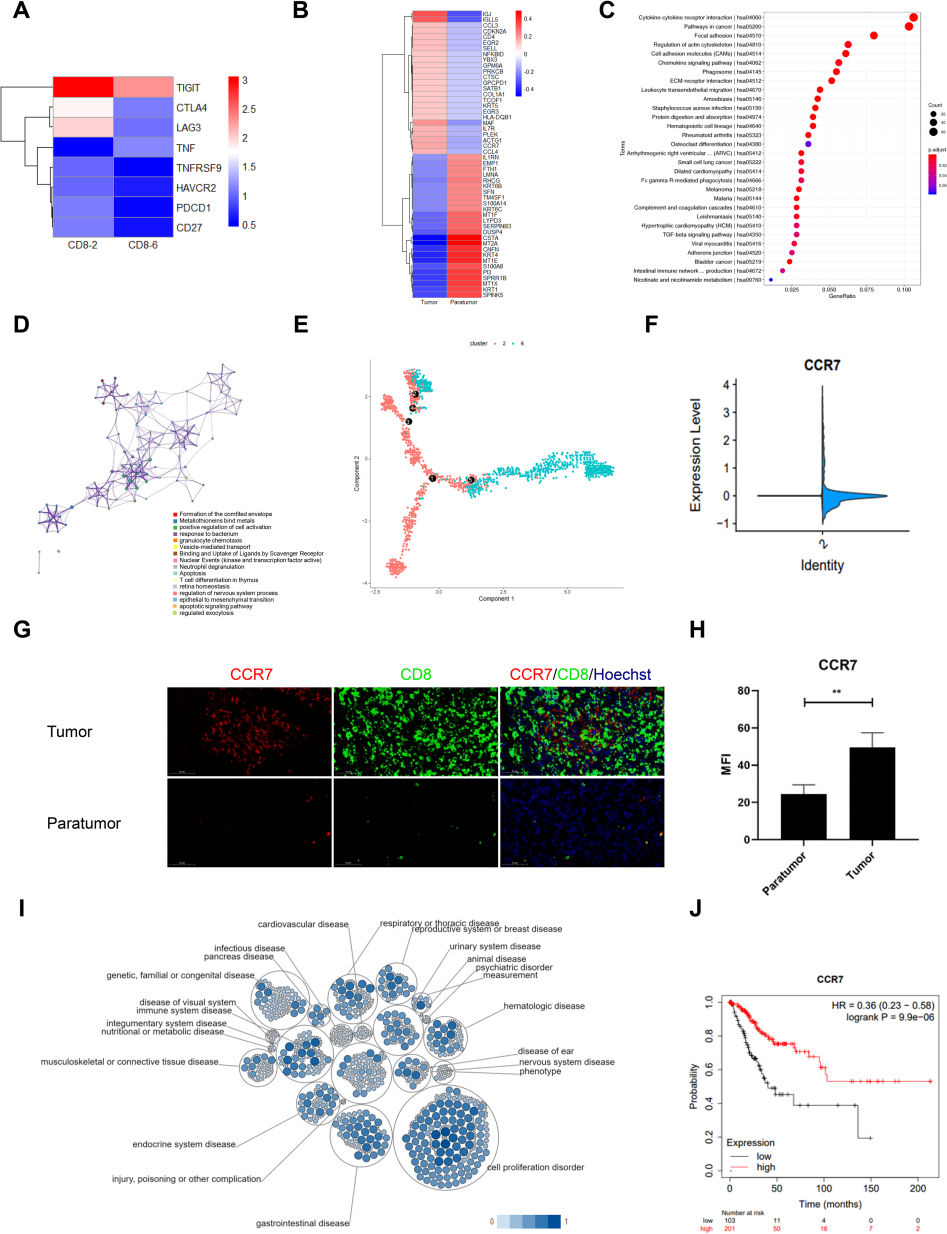
Gene Signature of CD8^+^ T Cells in Cervical Squamous Cell Carcinoma (CSCC). **(A)** Heatmap of inhibitory checkpoint expression in CD8^+^ T cell clusters 2 and 6 generated by Heml 1.0. **(B)** Top 50 differentially expressed genes (DEGs) in exhausted CD8^+^ T cells between tumor and paratumor tissues. **(C)** KEGG pathway analysis of DEGs in exhausted CD8^+^ T cells; circle size denotes gene count, color indicates adjusted p-value. **(D)** Reactome pathway enrichment via Metascape with spider plot visualization. **(E)** Trajectory analysis of CD8^+^ T cells in CSCC. **(F)** CCR7 expression comparison between tumor and paratumor tissues. **(G)** Immunofluorescence analysis of CCR7 and CD8 expression; scale bar, 50 μm. **(H)** Mean fluorescence intensity of CCR7 in tumor versus paratumor tissues (**p < 0.01). **(I)** Pathway and disease association of CCR7 analyzed using Open Targets. **(J)** Prognostic impact of CCR7 in CSCC assessed by KM-plotter.

Comprehensive profiling of CD8^+^ T cell subsets in CSCC revealed distinct molecular signatures and functional states. Cluster 2 exhibited marked upregulation of multiple inhibitory checkpoints linked to T cell exhaustion, including CTLA-4, PDCD1 (PD-1), HAVCR2 (TIM3), CD27, LAG3, TNFRSF9, and TIGIT (**Figure 3A**). Within these subsets, exhausted cluster 2 (CD8-2) showed higher TIM3 and LAG3 expression compared to cluster 6 (CD8-6), while PD-1 levels remained uniformly low across samples (**Supplementary Figure S1**). These results define a unique immune checkpoint profile characteristic of T cell exhaustion and emphasize the functional heterogeneity of CD8^+^ T cells in the tumor microenvironment. Accordingly, cluster 2 was designated as the exhausted CD8^+^ T cell population.

To investigate the distribution of exhausted CD8^+^ T cells in the tumor microenvironment, we conducted heat map analysis comparing gene expression profiles of tumor and paratumor tissues. Upregulated genes in tumor tissues included IGJ, IGLL5, CCL3, CDKN2A, CD4, EGR2, SELL, NFKBID, YBX3, GPM6A, PRKCB, CTSC, GPCPD1, SATB1, COL1A1, TCOF1, KRT5, EGR3, HLA-DQB1, MAF, IL7R, PLEK, ACTG1, CCR7, and CCL4. Conversely, downregulated genes were IL1RN, EMP1, FTH1, LMNA, RHCG, KRT6B, SFN, TM4SF1, S100A14, KRT6C, MT1F, LYPD3, SERPINB3, DUSP4, CSTA, MT2A, CNFN, KRT4, MT1E, S100A8, PI3, SPRR1B, MT1X, KRT1, and SPINK5 (**Figure 3B**). Pathway enrichment analysis indicated that the DEGs in tumor-infiltrating CD8^+^ T cells may be involved in several pathways, including cytokine-cytokine receptor interaction, cancer pathways, focal adhesion, regulation of the actin cytoskeleton, chemokine signaling, phagosome formation, ECM-receptor interaction, leukocyte transendothelial migration, amoebiasis, Staphylococcus aureus infection, protein digestion and absorption, hematopoietic cell lineage, and rheumatoid arthritis (**Figure 3C**). Furthermore, **Figure 3D** illustrates the interconnectivity of various biological processes through complex networks.

Using the Monocle algorithm, we reconstructed the developmental trajectory of CD8^+^ T cells during tumor progression, revealing a clear transition from naïve and effector states to exhausted CD8^+^ T cells and a final terminal state (**Figure 3E**, **Supplementary Figure S2A**). This progression reflects the typical evolution of T cell states within tumors and suggests a gradual onset of exhaustion correlating with tumor advancement and potentially poorer clinical outcomes (**Supplementary Figure S2E**). Pseudotime analysis identified 11 distinct cellular states along this trajectory, with principal component analysis (PCA) demonstrating clear separation and clustering reflective of transcriptional heterogeneity (**Supplementary Figures S3A, B**). State 1 was assigned as the root population representing the initial naïve CD8^+^ T cells. A heatmap of 50 differentially expressed genes (FDR < 0.01) highlighted distinct expression clusters aligned with divergent cell fates (**Supplementary Figure S3C**). Notably, genes such as CXCR4, DNAJB1, FCGBP, HSP90AA1, and HSP90AB1 displayed dynamic expression changes across pseudotime, clusters, and cellular states (**Supplementary Figures S3D-F**). Among these, FCGBP was highly expressed in cluster 6/state 8 cells, significantly exceeding levels in the other ten cellular states. The mechanisms and implications of FCGBP upregulation in this specific developmental state of cervical cancer CD8^+^ T cells warrant further investigation. Together, these findings reveal a complex regulatory landscape underlying CD8^+^ T cell differentiation and underscore the functional heterogeneity and transitional relationships among cell populations within the tumor microenvironment.

Our results demonstrated a significant increase in CCR7 expression in tumor tissues compared to paratumor tissues (**Figure 3F**, **Supplementary Figure S2B**). This was supported by immunofluorescence staining, which confirmed elevated CCR7 levels in tumor samples (**Figures 3G, H**, **Supplementary Figure S2C**). Data from the Tumor Immune Estimation Resource (TIMER) database revealed a positive correlation between CCR7 expression and CD8^+^ T cell abundance in cervical cancer (**Supplementary Figures S2D, G**). Furthermore, CCR7 expression exhibited a negative correlation with tumor purity and a strong positive correlation with CD8^+^ T cell infiltration levels (**Supplementary Figures S2E, F**).

To evaluate CCR7’s prognostic value in CSCC, we analyzed data from 304 patients in the TCGA database using the Kimplot tool. Open Targets analysis revealed CCR7’s prominent involvement in immune system diseases, especially within the reproductive system (**Figure 3I**). Importantly, analysis of TCGA data showed that elevated CCR7 expression was correlated with significantly improved overall survival (OS) (P = 9.9e-6; **Figure 3J**). These results suggest an association between CCR7 expression and CD8^+^ T cell infiltration in CSCC, indicating its potential as a prognostic biomarker that warrants further investigation.

### 3.4 Upregulation of CCL5 and its related pathways in tumor Tregs and inflammation regulation

**Figure 4:**
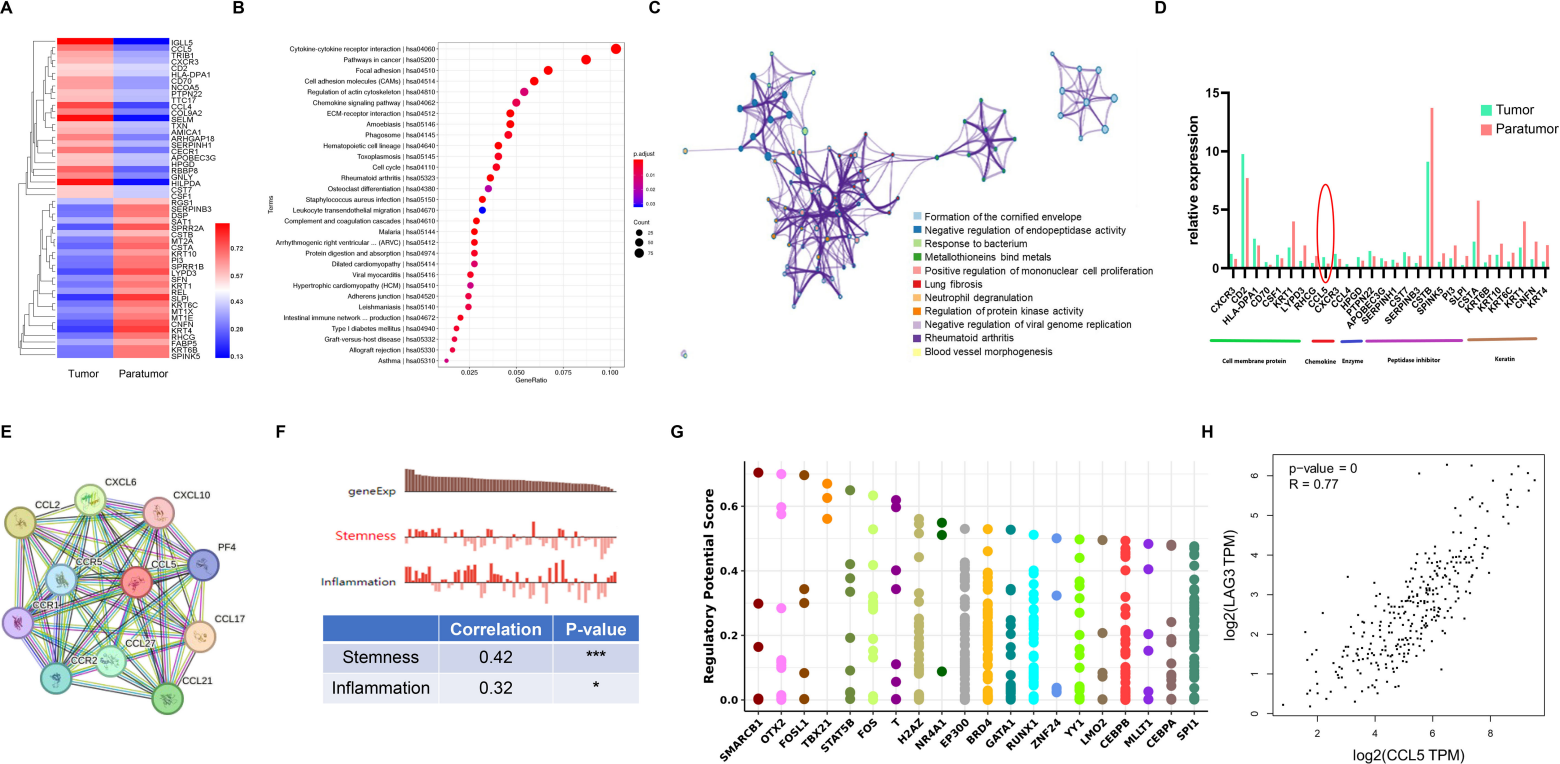
Treg Gene Signature in Cervical Squamous Cell Carcinoma (CSCC). **(A)** Heatmap of the top 50 differentially expressed genes (DEGs) in Tregs between tumor and paratumor tissues. **(B)** KEGG pathway analysis of Treg DEGs; circle size reflects gene count, color indicates adjusted p-value. **(C)** Reactome pathway enrichment via Metascape (https://metascape.org/gp/index.html) with spider plot visualization. **(D)** Expression profiles of key molecules in Tregs. **(E)** Protein interaction network of CCL5 mapped using STRING. **(F)** Functional characterization of Tregs analyzed with CancerSEA (http://biocc.hrbmu.edu.cn/CancerSEA/). **(G)** Identification of the top 20 differentially expressed transcription factors associated with CCL5 in cancers via Cistrome DB Toolkit (http://dbtoolkit.cistrome.org). **(H)** Correlation between CCL5 and LAG3 analyzed using GEPIA2 (http://gepia2.cancer-pku.cn/#index). .

Tumor-infiltrating Tregs analysis revealed upregulation of IGLL5, CCL5, TRIB1, CXCR3, CD2, HLA-DPA1, CD70, NCOA5, PTPN22, TTC17, CCL4, COL9A2, SELM, TXN, AMICA1, ARHGAP18, SERPINH1, CECR1, APOBEC3G, HPGD, RBBP8, GNLY, HILPDA, CST7 and CSF1 in tumors versus paratumor tissues. Conversely, RGS1, SERPINB3, DSP, SAT1, SPRR2A, CSTB, MT2A, CSTA, KRT10, PI3, SPRR1B, LYPD3, SFN, KRT1, REL, SLPI, KRT6C, MT1X, MT1E, CNFN, KRT4, RHCG, FABP5, KRT6B, and SPINK5 were downregulated (**Figure 4A**). Functional pathway analysis revealed that these genes were enriched in cytokine-cytokine interaction, pathways in cancer, focal adhesion, and cell adhesion molecules (CAMs) pathways (**Figure 4B**). The Reactome network illustrated their involvement across diverse biological processes (**Figure 4C**), emphasizing key cell membrane proteins, chemokines, enzymes, peptidase inhibitors, and keratin. Notably, the chemokine CCL5 was overexpressed in tumor-infiltrating Tregs (**Figure 4D**). STRING network analysis linked CCL5 with CCR5, CCL2, CCR2, CCR1, CXCL10, PF4, CCL17, CCL21, CCL27, and CXCL6 (**Figure 4E**). Single-cell analysis via CancerSEA indicated CCL5’s primary role in inflammation regulation (**Figure 4F**). To elucidate CCL5’s transcriptional regulation, we identified the top 20 cancer-associated transcription factors using the Cistrome DB Toolkit (**Figure 4G**). Furthermore, GEPIA2 analysis demonstrated a positive correlation between CCL5 and LAG3 (**Figure 4H**).

### 3.5 Gene signatures of B cells and functional pathway dissection

**Figure 5:**
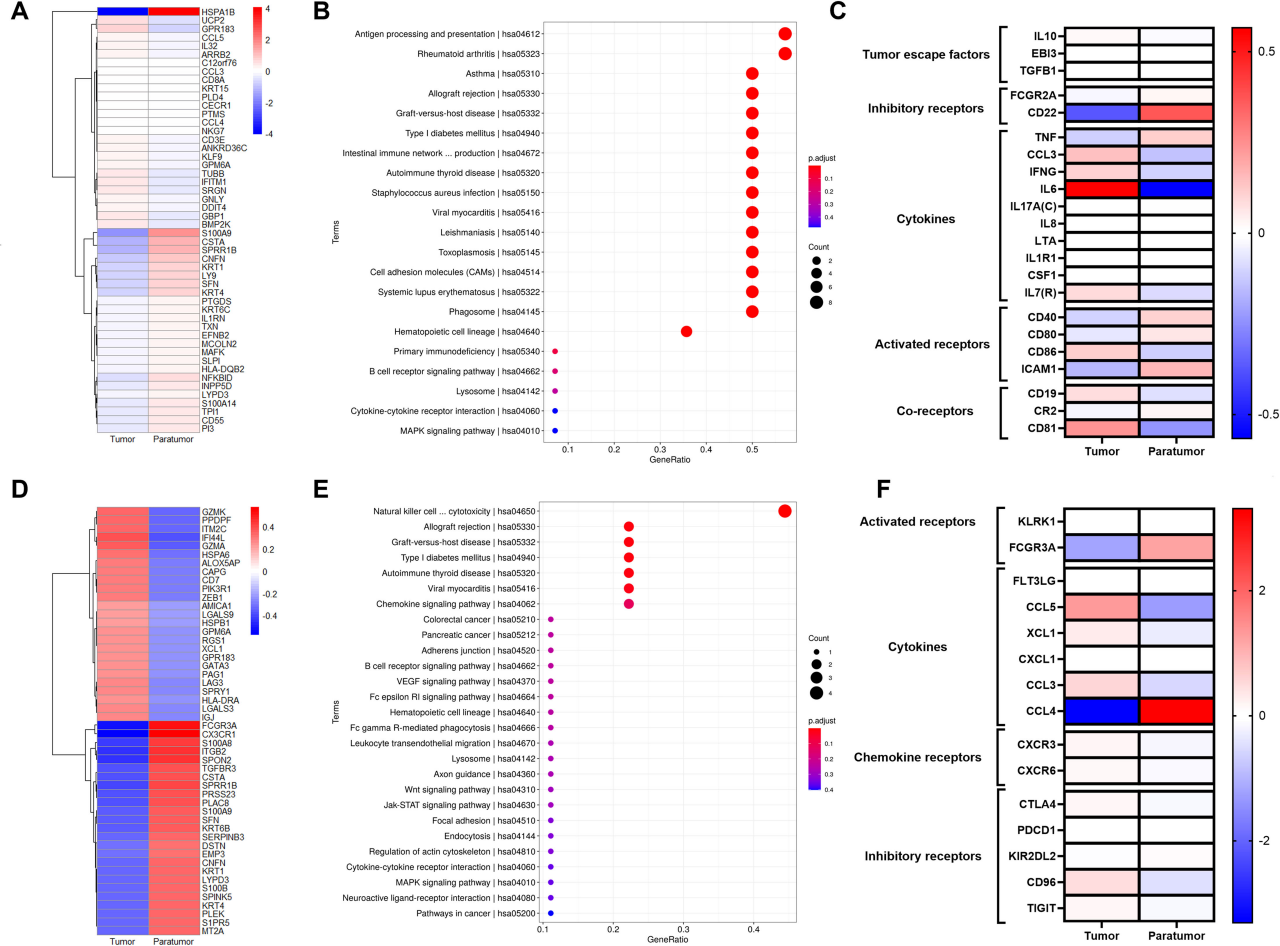
Gene Signatures of B Cells and NK/T Cells in Cervical Squamous Cell Carcinoma (CSCC). **(A)** Heatmap of the top 50 differentially expressed genes (DEGs)in B cells comparing tumor and paratumor tissues. **(B)** KEGG pathway analysis of B cell DEGs; circle size denotes gene count, color reflects adjusted p-value. **(C)** Comparison of functional molecule levels in B cells between tumor and paratumor tissues. **(D)** Heatmap showing the top 50 DEGs in NK/T cells between tumor and paratumor tissues. **(E)** KEGG pathway analysis of NK/T cell DEGs; circle size indicates gene number, color shows adjusted p-value. **(F)** Expression profiles of functional molecules in NK/T cells.

We identified DEGs in B cell subsets between tumor and paratumor tissues. Tumor tissues exhibited upregulation of genes including UCP2, GPR183, CCL5, IL32, ARRB2, C12orf76, CCL3, CD8A, KRT15, PLD4, CECR1, PTMS, CCL4, NKG7, CD3E, ANKRD36C, KLF9, GPM6A, TUBB, IFITM1, SRGN, GNLY, DDIT4, GBP1, and BMP2K, while genes such as HSPA1B, S100A9, CSTA, SPRR1B, CNFN, KRT1, LY9, SFN, KRT4, PTGDS, KRT6C, IL1RN, TXN, EFNB2, MCOLN2, MAFK, SLPI, HLA-DQB2, NFKBID, INPP5D, LYPD3, S100A14, TPI1, CD55, and PI3 were downregulated (**Figure 5A**). Pathway analysis linked both up- and downregulated genes to antigen processing and presentation pathways (**Figure 5B**). Heatmap comparisons emphasized B cells’ immunoregulatory roles, with elevated escape factor IL10 and reduced inhibitory factors FCGR2A and CD22. Cytokines such as CCL3, IFNG, LTA, and IL7R were increased. Receptor analysis showed divergent patterns: CD86 and co-receptor CD81 were upregulated, whereas ICAM1 and CR2 were downregulated (**Figure 5C**, **Supplementary Table S5**).

### 3.6 Elevated inhibitory receptor expression and reduced activating receptor expression in NK/T cells

NK/T cells mediate host defense via cytokine secretion and cytolytic activity against tumor cells. We observed distinct gene expression profiles in NK/T cells from tumor versus paratumor tissues. Tumor-infiltrating NK/T cells upregulated genes including GZMK, PPDPF, ITM2C, IF144L, GZMA, HSPA6, ALOX5AP, CAPG, CD7, PIK3R1, ZEB1, AMICA1, LGALS9, HSPB1, GPM6A, RGS1, XCL1, GPR183, GATA3, PAG1, LAG3, SPRY1, HLA-DRA, LGALS3, and IGJ, while genes such as FCGR3A, CX3CR1, S100A8, ITGB2, SPON2, TGFBR3, CSTA, SPRR1B, PRSS23, PLAC8, S100A9, SFN, KRT6B, SERPINB3, DSTN, EMP3, CNFN, KRT1, LYPD3, S100B, SPINK5, KRT4, PLEK, S1PR5, and MT2A were downregulated (**Figure 5D**). Functional pathway analysis implicated these genes in Natural killer cell mediated cytotoxicity, Allograft rejection, Graft-versus-host disease, Type I diabetes mellitus, Autoimmune thyroid disease, Viral myocarditis, Chemokine signaling pathway (**Figure 5E**).

NK/T cells in tumor tissues displayed elevated expression of inhibitory receptors CTLA4, PDCD1, CD96, and TIGIT, alongside reduced expression of the activating receptor FCGR3A compared to paratumor tissues. Tumor-infiltrating NK/T cells also secreted higher levels of cytokine CCL3 and exhibited increased expression of chemokine receptors CXCR3 and CXCR6 (**Figure 5F**, **Supplementary Table S6**).

### 3.7 Characterization of single-cell expression profiles in pDC cells

**Figure 6:**
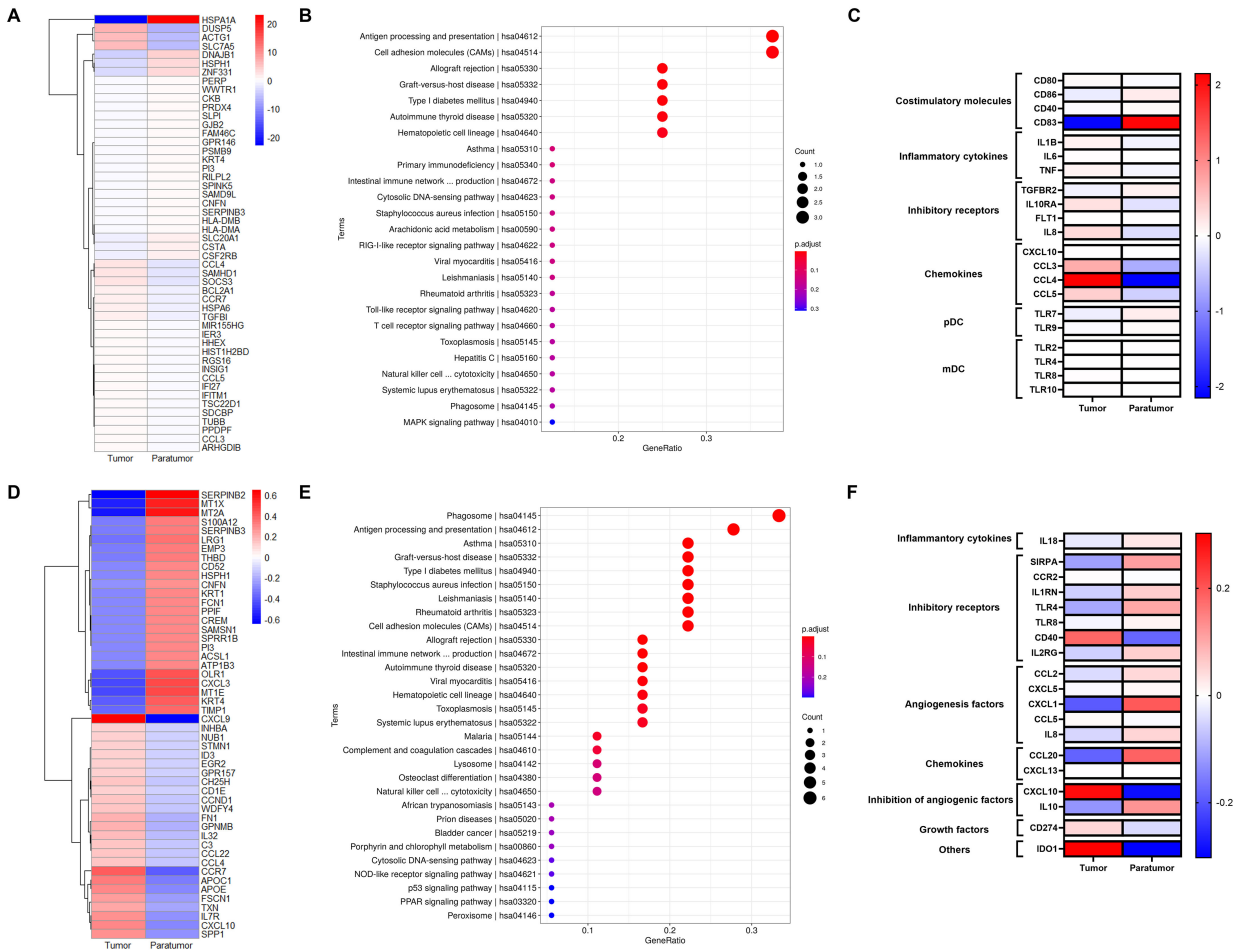
Gene Signatures of pDCs and Macrophages in Cervical Squamous Cell Carcinoma (CSCC). **(A)** Heatmap showing the top 50 differentially expressed genes (DEGs)in pDCs between tumor and paratumor tissues. **(B)** KEGG pathway analysis of pDC DEGs; circle size represents gene count, color indicates adjusted p-value. **(C)** Expression analysis of functional molecules in pDCs comparing tumor and paratumor tissues. **(D)** Heatmap of the top 50 DEGs in macrophages between tumor and paratumor tissues. **(E)** KEGG pathway analysis of macrophage DEGs; circle size denotes gene number, color reflects adjusted p-value. **(F)** Comparison of functional molecule expression in macrophages between tumor and paratumor tissues.

Dendritic cells (DCs), key regulators of adaptive immunity, are critical for T cell-mediated cancer responses. Differentially expressed genes (DEGs) in the DC cluster are primarily enriched in antigen processing and presentation pathways (**Figures 6A, B**).

pDCs, key producers of type I interferons (IFN-I), enhance anti-tumor immunity by directly affecting tumor and immune cells. They selectively express TLR7 and TLR9, producing large amounts of IFN-I in response to single-stranded viral RNA and DNA (**Figure 6C**). Our data show that tumor-infiltrating pDCs upregulate inhibitory receptors IL10RA and IL8 compared to paratumor tissues. Similar to B cells, pDCs display distinct expression patterns of activating receptors, cytokines, chemokine receptors, and inhibitory receptors in tumor versus paratumor tissues. They also secrete elevated levels of CXCL10, CCL3, CCL4, and CCL5 in tumors (**Figure 6C**, **Supplementary Table S7**). These findings underscore the complexity of pDC biology and suggest that multiple complementary approaches will be required to optimize DC-based therapies for cervical cancer.

### 3.8 Gene signature of macrophages in CSCC

Macrophages, crucial components of the tumor microenvironment, can either promote or inhibit tumorigenesis and metastasis depending on their activation state. Differentially expressed genes (DEGs) in the macrophage cluster (**Figure 6D**) were mainly enriched in phagosome activity and antigen processing and presentation pathways (**Figure 6E**). To further define macrophage function, we analyzed key inflammatory cytokines, inhibitory receptors, angiogenic and anti-angiogenic factors, chemokines, and growth factors in tumor and paratumor tissues, visualized by heatmaps. Tumor-associated macrophages showed increased expression of CCL5, CXCL13, CXCL10, CD274, and IDO1, alongside decreased expression of SIRPA, TLR4, TLR8, CCL2, CXCL5, CXCL1, IL8, CCL20, and IL10 (**Figure 6F**, **Supplementary Table S8**). Notably, IL-10 expression was elevated in tumor tissues. These results indicate a mixed M1/M2 macrophage phenotype within the tumor microenvironment.

## 4 Discussion

Cervical cancer, a common malignancy in women, is driven by cancer cell heterogeneity and intercellular interactions within the TME (18, 31). scRNA-seq has become crucial for delineating the cellular makeup of the cervical cancer TME and defining molecular subtypes. Prior studies identified four molecular subtypes—hypoxia (S-H), proliferation (S-P), differentiation (S-D), and immunoactive (S-I)—with the S-I subtype associated with the best overall survival (31), highlighting the TME’s significance. Investigations into immune cells within the TME reveal that tumor-infiltrating immune cells (TICs) serve as valuable biomarkers for chemotherapy response (16, 32, 33). Unlike previous work, this study offers a comprehensive single-cell characterization of the cervical cancer microenvironment by comparing tumor tissue with paratumor tissue from CSCC patients. The identified immune-related differential genes may suggest potential prognostic markers and therapeutic targets that require further validation. This study is based on a small sample size (n=3) with results that are exploratory and hypothesis-generating; although partially validated using database data, further research is needed to confirm their reliability.

The TME comprises immune and stromal cells, chemokines, cytokines, and the extracellular matrix components (34). We found increased numbers and proportions of CD8^+^ T cells in tumor versus paratumor tissues. These CD8^+^ T cells exhibit functional heterogeneity, transitioning along a differentiation trajectory from naïve and effector states to an exhausted phenotype characterized by impaired cytokine secretion and proliferative capacity, limiting tumor control (35). Consistent with this, single-cell analyses reveal T and NK cell enrichment in tumors with a shift from cytotoxic to exhausted states (36). CD8^+^ T cell infiltration closely correlates with cervical cancer prognosis, with related genes potentially driving disease progression (37). CCR7, a G protein-coupled receptor involved in inhibiting apoptosis of mature dendritic cells (38), was significantly upregulated in CD8^+^ T cells, with higher CCR7 levels linked to favorable outcomes, indicating a positive immune regulatory role. CCR7 expression exhibits a dual role in cancer biology: while its presence on cancer cells facilitates tumor progression, expression on immune cells enhances antitumor responses (39–42). In CSCC, CCR7 levels may serve as a prognostic biomarker and reflect a shift in the tumor microenvironment (TME) from immune-dominance to metabolic activation (43). Notably, CCR7^+^ CD8^+^ T cells constitute an apoptosis-resistant subset (44) and their abundance correlates with improved survival in advanced colorectal cancer (45). Additionally, CCR7 regulates hepatic CD8^+^ T cell homeostasis and confers protection against acute liver injury (46). Consistent with these findings, increased infiltration of CD8^+^ lymphocytes is generally associated with favorable prognosis following adjuvant chemotherapy in cervical cancer (47). Our data reveal a positive correlation between CCR7^+^ CD8^+^ T cell expression and overall survival, suggesting CCR7 as a potential immunoregulatory molecule and a preliminary therapeutic target in cervical cancer that merits further investigation.

We observed that both the number and proportion of Tregs were elevated in cervical cancer tissues compared to paratumor normal tissues, consistent with findings by Wu et al. (48). As immunosuppressive cells, Treg upregulation is associated with poor prognosis in various cancers (49). For instance, Tregs promote angiogenesis in ovarian cancer (50) and facilitate immune evasion by suppressing effector T cells through anti-inflammatory effects (51). Further analysis revealed enrichment of CCL5 within the cytokine–cytokine receptor interaction pathway in Tregs, with CCL5 levels significantly higher in tumor tissues than in paratumor tissues. A prior study demonstrated that increased Treg numbers correlate with the expression of CCR5 and its ligand CCL5 (52). Wang et al. demonstrated that the CCL5/CCR5 axis facilitates Treg infiltration into the tumor microenvironment, with CCL5 expression positively correlating with and being directly activated at the transcriptional level by c-Foxp3 (53). CCL5 promotes the migration of Foxp3^high^ Tregs from peripheral blood to the tumor microenvironment via the CCR5 receptor, and the ratio of peripheral Foxp3^high^ Tregs to CD4^+^ T cells is significantly associated with intratumoral Treg levels, suggesting its potential as a peripheral biomarker (54, 55). In breast cancer, CCL5 enhances the Treg/CD4^+^CCR5^+^ cell ratio through CCR5, thereby promoting lymph node metastasis (55).

We detected B cells in both cervical cancer tumor tissues and paratumor normal tissues, with comparable proportions relative to total cell counts. Zou et al. reported similar findings in their study of immune cell infiltration in cervical cancer, noting that B cell infiltration was not associated with survival outcomes (56). The impact of B cell infiltration on cervical cancer prognosis remains unclear. It is established that plasma cells, by producing antibodies, contribute positively to antigen clearance and inhibition of tumor progression, whereas regulatory B cells (Bregs), characterized by IL-10 secretion, suppress antitumor immunity (57). Bregs have been shown to promote tumor growth by inhibiting T cell responses through IL-10 secretion (58). Zhao et al. reviewed the dual roles of B cells in tumor immunity, emphasizing the critical function of cytokines in mediating these effects (59). Based on these insights, we speculate that B cells in cervical cancer tissues may produce IL-10 to modulate tumor development, although the precise mechanisms warrant further investigation.

We further analyzed key inflammatory cytokines, inhibitory and activating receptors, angiogenic factors, chemokines, angiogenesis inhibitors, growth factors, and other relevant molecules within NK/T cells, dendritic cells (DCs), and macrophages, presenting the data as a heatmap. Notably, genes significantly upregulated in NK/T cells were enriched in NK cell-mediated cytotoxicity pathways, while the chemokine CXCL1 was markedly downregulated in cervical cancer. Downregulation of CXCL1 reduces cellular viability, impairs proliferation, diminishes migratory capacity, and promotes apoptosis of cervical cancer cells (60). Conversely, high CXCL1 expression in cervical cancer tissues is significantly associated with poor clinical survival, potentially due to ERK1/2 pathway activation driving tumor malignancy (61).

We found that genes significantly upregulated in plasmacytoid dendritic cells (pDCs) were enriched in Antigen processing and presentation and Cell adhesion molecule (CAM) pathways. The chemokines CCL3, CCL4, and CCL5 were markedly elevated in cervical cancer. Previous studies have demonstrated that CCL3, CCL4, and CCL5 recruit pro-tumor Ly6C^high^ monocytes or M2 macrophages via the CCR5 receptor, thereby suppressing antitumor immunity (62, 63). Additionally, we observed significant upregulation of the inhibitory receptor CXCL10 in cervical cancer tissues. CXCL10 levels were inversely correlated with vascular endothelial growth factor (VEGF) in cervical cancer, indicating its capacity to inhibit VEGF-mediated angiogenesis. As a prognostic marker for CSCC, CXCL10 may suppress tumor growth by modulating microvascular formation and regulating the expression of HPV oncogenes E6 and E7 (64).

We observed that genes significantly upregulated in macrophages within cervical cancer were enriched in Phagosome as well as Antigen processing and presentation pathways. Macrophages in cervical cancer exhibited a mixed M1/M2 phenotype, gradually shifting from M1 to M2 as cancer cells infiltrated from tumor to paratumor tissues. In the peritumoral region, activated M2 macrophages exert immunosuppressive functions that facilitate disease progression.

This study has several limitations that should be acknowledged. First, the single-cell RNA sequencing was performed on a small cohort of three patients, which may limit the generalizability of our findings. To mitigate this, we compared our results with data from public databases such as TCGA-CESC, and we intend to expand the sample size in future studies to further validate and strengthen our conclusions. Second, confirmation of co-expression and spatial distribution of CD8, CCR7, and established exhaustion markers (e.g., PD-1, TIM3, LAG3) through multiplex immunofluorescence co-staining remains to be completed. Third, while CCR7 emerged as a prognostic marker and CCL5 as a potential recruiter of regulatory T cells, functional studies—such as CCR7 knockdown or CCL5 blockade—are necessary to clarify their causal roles and deepen mechanistic insight. Additionally, analysis of cell-cell communication networks within the tumor microenvironment, for instance using tools like CellPhoneDB to map ligand-receptor interactions, has yet to be conducted. Addressing these gaps will be essential in future work to fully elucidate the immunological landscape of cervical cancer.

Although TME-targeted strategies have expanded therapeutic options for cancer, the immune landscape of cervical cancer remains incompletely understood. We performed single-cell profiling of primary cervical tumors and paratumor normal tissues, revealing distinct TME characteristics between these compartments. Cytokines play crucial roles in regulating key immune cells within the TME, including CD8^+^ T cells, Tregs, and B cells. Given their capacity to initiate antitumor T cell immunity, NK/T cells, pDCs, and macrophages are central to anticancer immunotherapy. Moving forward, novel immunotherapeutic interventions for cervical cancer should focus on enhancing the function of tumor-associated NK/T cells, pDCs, and macrophages to improve patient outcomes. These findings offer preliminary insights into cervical cancer biology, which may guide future exploratory research on therapeutic targets.

## Data Availability

The original contributions presented in the study are publicly available. This data can be found here: NCBI GEO repository, accession number GSE308792.

## Supplementary material

Supplementary Figure 1 Immune checkpoint molecule expression in tumor versus paratumor tissues and CD8^+^ T cell subpopulations. **(A)** Heatmap showing differential expression of CCR7, TM3, PD1, and LAG3 between tumor and paratumor tissues. Hierarchical clustering highlights distinct expression patterns, reflecting their potential roles in the tumor microenvironment. **(B)** Heatmap of immune checkpoint expression in two CD8^+^ T cell subpopulations: exhausted cluster 2 (CD8_2) and cluster 6 (CD8_6). Clustering reveals elevated checkpoint expression in exhausted CD8^+^ T cells, indicating functional heterogeneity within the tumor milieu.

Supplementary Figure 2 CCR7 Correlates with CD8^+^ T Cells in Cervical Squamous Cell Carcinoma (CSCC). **(A)** Pseudotime reconstruction of CD8^+^ T cells (clusters 2 and 6) developmental trajectory. **(B)** T-distributed stochastic neighbor embedding (TSNE) analysis of CCR7 expression in tumor versus paratumor tissues. **(C)** Immunohistochemical images of CCR7 in tumor and paratumor tissues from The Human Protein Atlas (https://www.proteinatlas.org/) ). **(D)** Tumor Immune Estimation Resource (TIMER) database (http://timer.cistrome.org/) analysis of CCR7 correlation with immune cells in cervical cancer. **(E)** High CCR7 and CD8^+^ T cell levels associate with improved cervical cancer survival. **(F)** CCR7 expression negatively correlates with tumor purity but positively with CD8^+^ T cell infiltration (http://timer.cistrome.org/). **(G)** CCR7 expression correlates with B cells, CD8^+^ and CD4^+^ T cells, macrophages, neutrophils, and dendritic cells (http://timer.cistrome.org/).

Supplementary Figure 3 Monocle pseudotime analysis and gene expression dynamics in single-cell RNA sequencing data. **(A)** PCA depicting cell distribution by pseudotime across 11 cellular states, with nodes marking key trajectory points. **(B)** Zoomed PCA views illustrating cell clustering and heterogeneity within each state. **(C)** Heatmap of 50 differentially expressed genes along pseudotime, organized by two cell fate branches and six expression clusters. **(D)** Gene expression trends of CXCR4, DNAJB1, FCGBP, HSP90AA1, and HSP90AB1 across pseudotime. **(E)** Expression variation of these genes across distinct cell clusters. **(F)** Dynamic expression of marker genes across cellular states, highlighting transcriptional changes during cell differentiation.
