# Supplementary figures and images for: Profiling of immune cell subsets and functional characteristics of cervical cancer based on single cell RNA sequencing
Source: Front Immunol. 2025 Sep 25;16:1658705. doi: 10.3389/fimmu.2025.1658705 (PMC12508775; doi:10.3389/fimmu.2025.1658705)

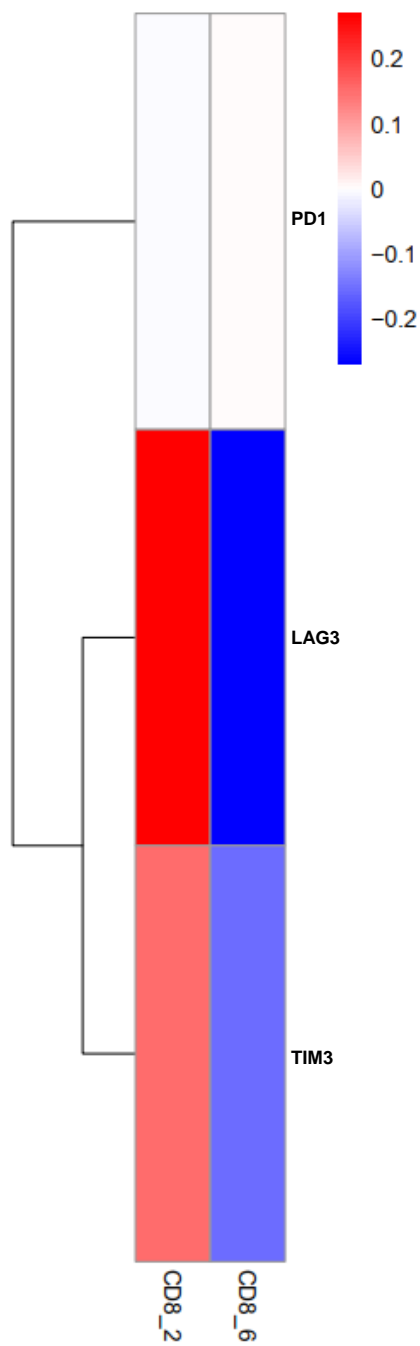

Supplement: Supplementary Figure 1 — Immune checkpoint molecule expression in tumor versus paratumor tissues and CD8+ T cell subpopulations. (A) Heatmap showing differential expression of CCR7, TM3, PD1, and LAG3 between tumor and paratumor tissues. Hierarchical clustering highlights distinct expression patterns, reflecting their potential roles in the tumor microenvironment. (B) Heatmap of immune checkpoint expression in two CD8+ T cell subpopulations: exhausted cluster 2 (CD8_2) and cluster 6 (CD8_6). Clustering reveals elevated checkpoint expression in exhausted CD8+ T cells, indicating functional heterogeneity within the tumor milieu. [file DataSheet1.pdf]

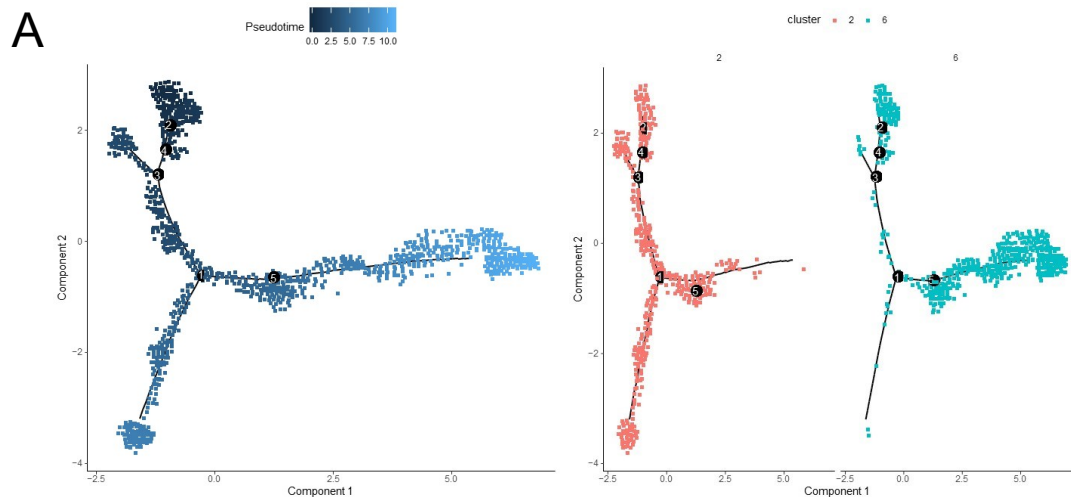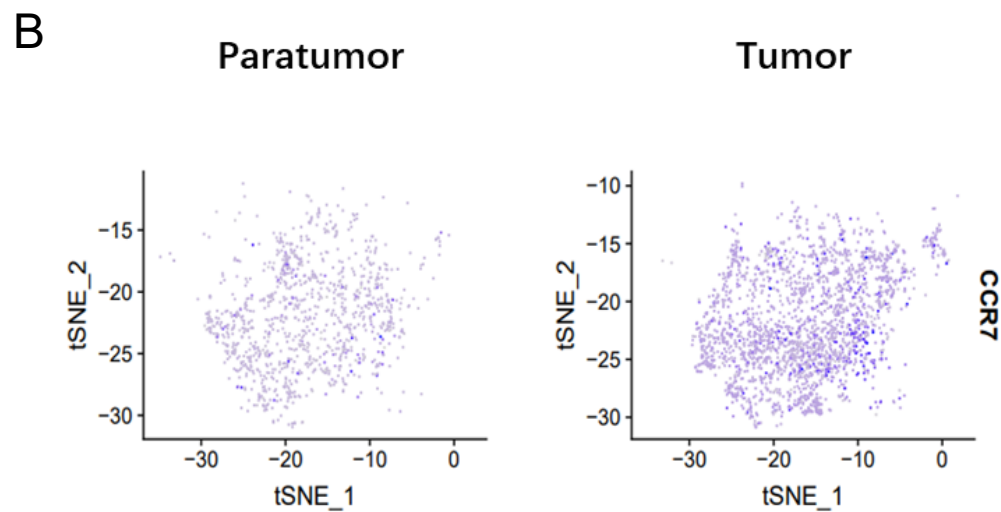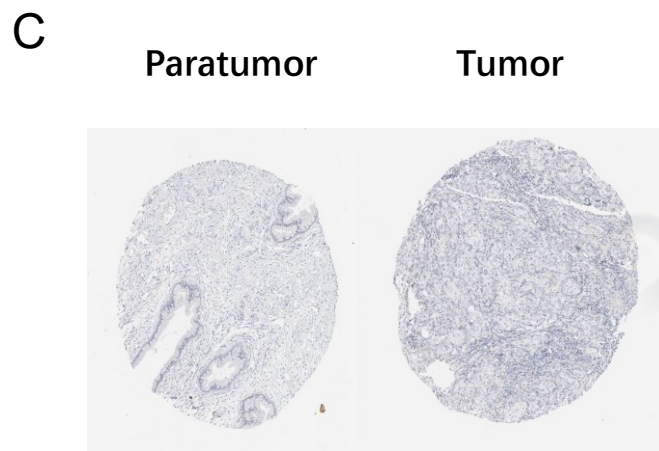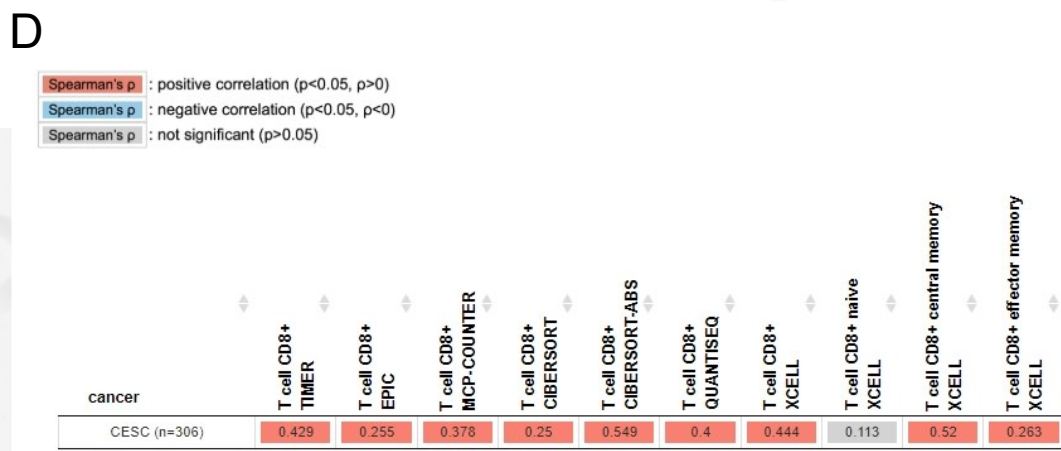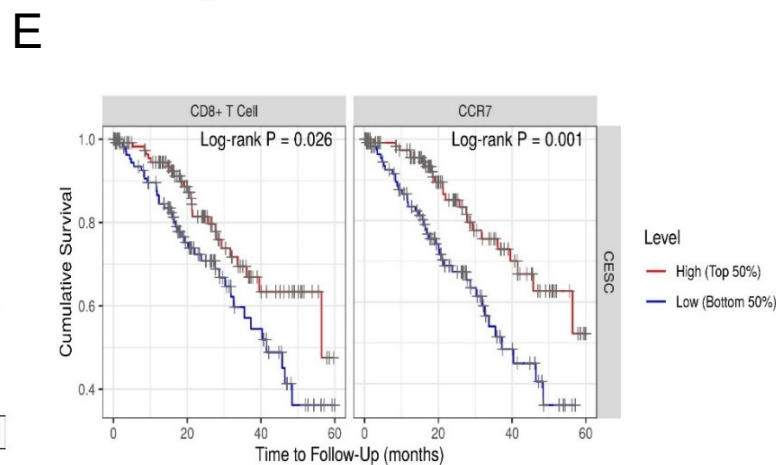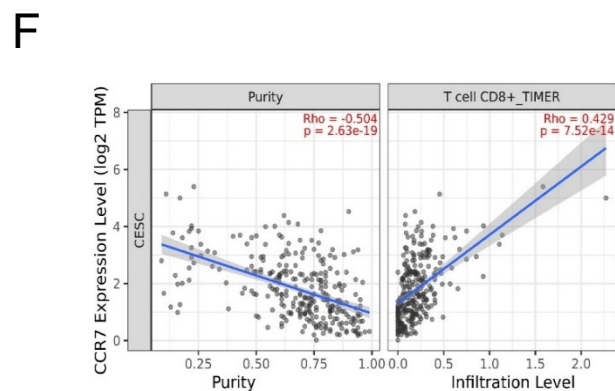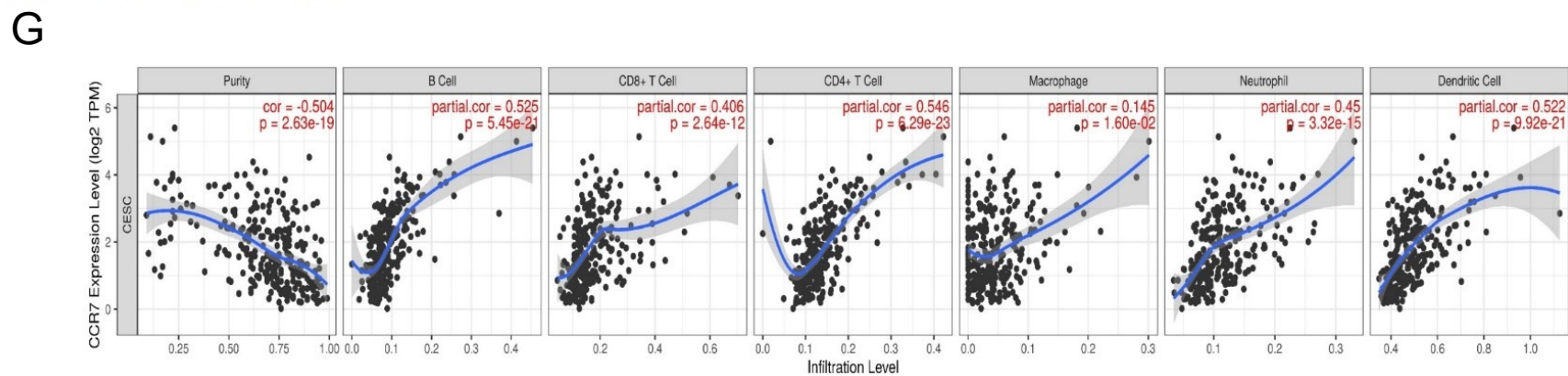

Supplement: Supplementary Figure 2 — CCR7 Correlates with CD8+ T Cells in Cervical Squamous Cell Carcinoma (CSCC). (A) Pseudotime reconstruction of CD8+ T cells (clusters 2 and 6) developmental trajectory. (B) T-distributed stochastic neighbor embedding (TSNE) analysis of CCR7 expression in tumor versus paratumor tissues. (C) Immunohistochemical images of CCR7 in tumor and paratumor tissues from The Human Protein Atlas (https://www.proteinatlas.org/) ). (D) Tumor Immune Estimation Resource (TIMER) database (http://timer.cistrome.org/) analysis of CCR7 correlation with immune cells in cervical cancer. (E) High CCR7 and CD8+ T cell levels associate with improved cervical cancer survival. (F) CCR7 expression negatively correlates with tumor purity but positively with CD8+ T cell infiltration (http://timer.cistrome.org/). (G) CCR7 expression correlates with B cells, CD8+ and CD4+ T cells, macrophages, neutrophils, and dendritic cells (http://timer.cistrome.org/). [file DataSheet2.pdf]

A

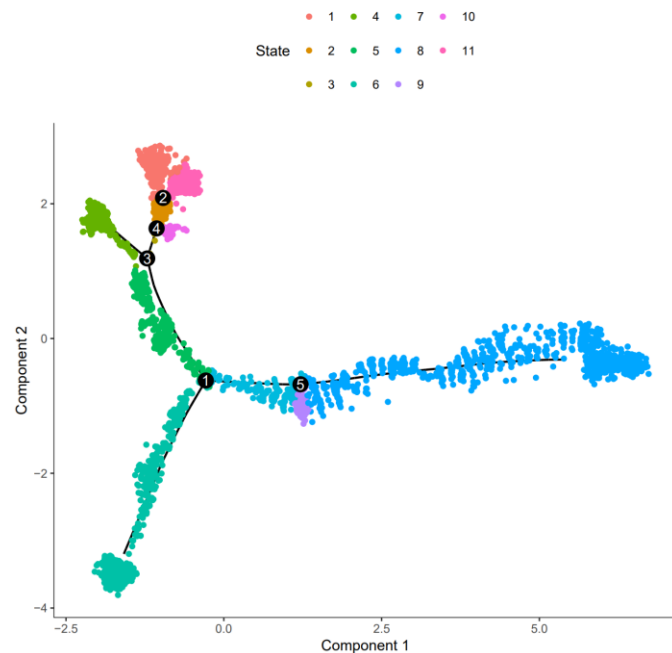

B

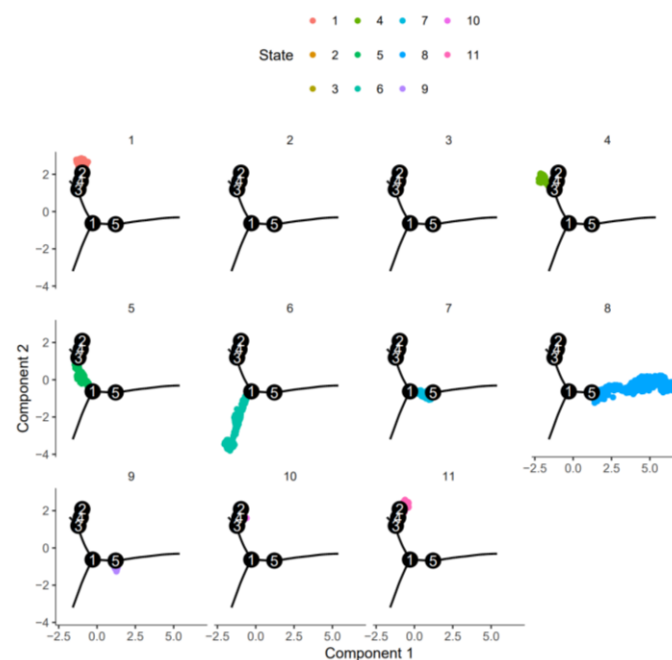

C

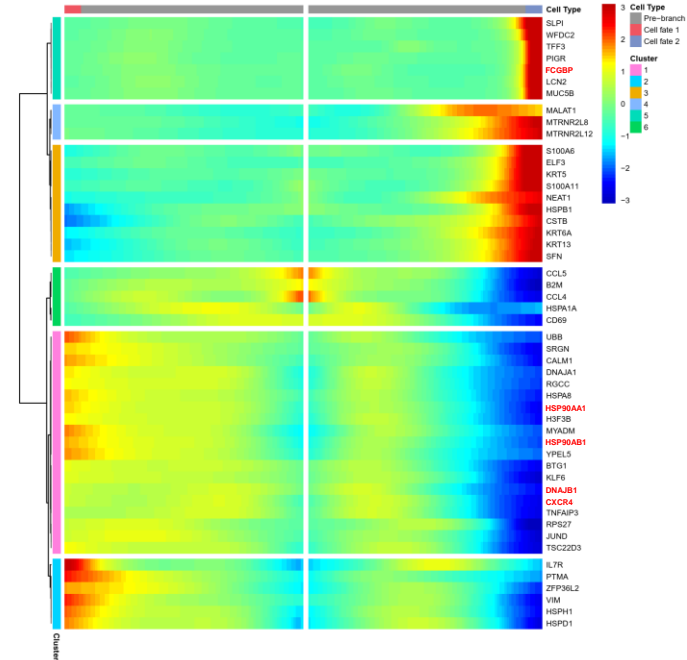

D

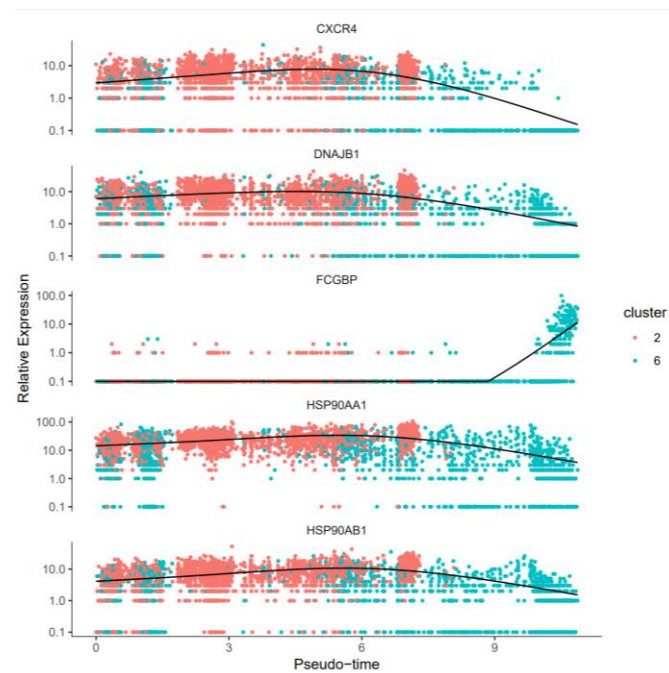

E

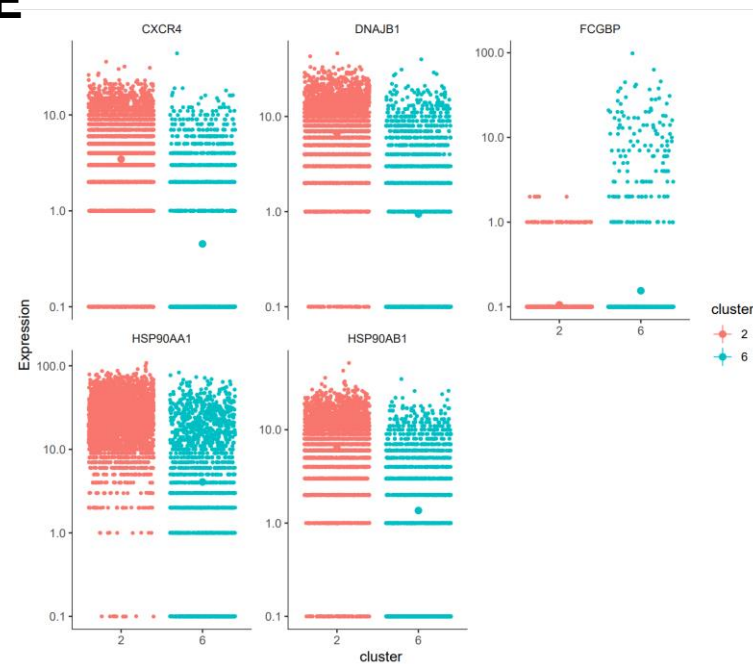

F

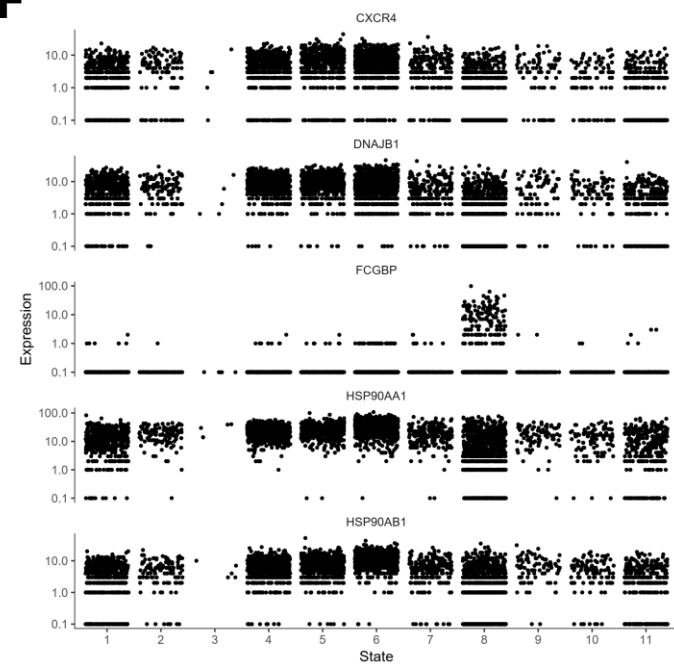

Supplement: Supplementary Figure 3 — Monocle pseudotime analysis and gene expression dynamics in single-cell RNA sequencing data. (A) PCA depicting cell distribution by pseudotime across 11 cellular states, with nodes marking key trajectory points. (B) Zoomed PCA views illustrating cell clustering and heterogeneity within each state. (C) Heatmap of 50 differentially expressed genes along pseudotime, organized by two cell fate branches and six expression clusters. (D) Gene expression trends of CXCR4, DNAJB1, FCGBP, HSP90AA1, and HSP90AB1 across pseudotime. (E) Expression variation of these genes across distinct cell clusters. (F) Dynamic expression of marker genes across cellular states, highlighting transcriptional changes during cell differentiation. [file DataSheet3.pdf]
